# The role of Ugandan District Hospital orthopedic units in the care of vulnerable road users: a cross-sectional study

**DOI:** 10.1186/s40621-016-0092-5

**Published:** 2016-12-05

**Authors:** Dan K. Kisitu, Lauren E. Eyler, Isaac Kajja, Gonzaga Waiswa, Titus Beyeza, David R. Ragland, Isabelle Feldhaus, Catherine Juillard, Rochelle A. Dicker

**Affiliations:** 1 Department of Surgery, Mbarara University of Science and Technology, P.O. Box 1410, Mbarara, Uganda; 2 Center for Global Surgical Studies, San Francisco General Hospital, University of California, Box 0807, San Francisco, CA 94143-0807 USA; 3 Department of Orthopaedics, Makerere University College of Health Sciences, P.O. Box 8062, Kampala, Uganda; 4 Mulago National Referral and Teaching Hospital, P.O. Box 7051, Kampala, Uganda; 5 Safe Transportation Research and Education Center (SafeTREC), School of Public Health, University of California, 2614 Dwight Way, Berkeley, CA 94720 USA

**Keywords:** Injury, Epidemiology, Less motorized countries, Road users

## Abstract

**Background:**

Musculoskeletal injuries are a common cause of morbidity after road traffic injury (RTI) in motorizing countries. District hospitals provide front-line orthopedic care in Uganda and other sub-Saharan African nations. Improving care at the district hospital level is an important component of the World Health Organization’s strategy for surgical and trauma systems strengthening, but the data necessary to inform RTI safety and care initiatives has previously been insufficient at the district hospital level. The objective of this study was to provide data on the patient population and patterns of musculoskeletal injury caused by RTI at Ugandan district hospitals.

**Methods:**

In this cross-sectional study, all patients with musculoskeletal injuries identified on x-ray presenting to three Ugandan district hospitals from October 2013 to January 2014 were interviewed and examined to obtain data on patient demographics and injury context by road user category. This manuscript is a sub-group analysis of RTI victims from a broader dataset of all musculoskeletal injuries.

**Results:**

Vulnerable road users comprised 92 % of musculoskeletal RTI patients, with 49 % (95 % CI 41–57 %) pedestrians, 41 % (95 % CI 33–49 %) motorcyclists, and 2 % (95 % CI 0–4 %) cyclists. Commonly injured subgroups included student pedestrians (33 % (95 % CI 22–44 %) of pedestrians) and motorcyclists with less than a post-secondary education (74 % (95 % CI 63–85 %) of motorcyclists). The morning hours were the most common time of injury for all RTI patients (37 %%; 95 % CI 30–44 %) and motorcyclists (46 %; 95 % CI 34–58 %), while pedestrians were most commonly injured in the evening (32 %; 95 % CI 21–43 %).

**Conclusions:**

By demonstrating commonly injured demographic groups and high frequency times of day for injury, this surveillance study of musculoskeletal RTI suggests targeted avenues for future road safety research in the districts of Uganda. Compared with previous studies from the capital of Uganda, these results suggest that Ugandan district hospitals care for a disproportionate share of vulnerable road users, a discrepancy which may pertain to other sub-Saharan African nations, as well. Strengthening district hospital orthopedic care should be considered a priority of strategies aimed at improving outcomes for these vulnerable groups.

## Background

Road traffic injuries (RTI) are a major and growing cause of morbidity and mortality in low- and middle-income countries (LMICs). Despite having far fewer registered motorized vehicles than high-income countries, LMICs account for 90 % of the world’s disability-adjusted life years (DALYs) due to RTI (Peden et al. 2004). Africa has the highest RTI fatality rate at 24.1 RTI deaths per 100,000 people per year, as compared to the Americas and Europe at 16.1 and 10.3 RTI deaths per 100,000 people per year, respectively (Peden et al. 2004). RTI rates are increasing dramatically in many sub-Saharan African nations, with one Kenyan study estimating a four-fold increase in RTI from the 1970s to 2000 (Odero et al. 2003), and a Tanzanian study estimating a 44 % increase in RTI from 1990 to 2000 (Museru et al. 2002). Among RTI victims, musculoskeletal injuries are common and debilitating. Studies from Uganda (Naddumba 2004; Galukande et al. 2009), Tanzania (Chalya et al. 2012), Nigeria (Okoro & Ohadugha 2010; Eluwa et al. 2010; Akinpelu et al. 2007), and Cameroon (McGreevy et al. 2014; Juillard et al. 2014) found that extremity injuries were the most common type of injury among patients presenting for care after RTI. As RTI rates increase, so does the importance of providing quality musculoskeletal care throughout sub-Saharan Africa.

The World Health Organization (WHO) has emphasized the importance of protecting vulnerable road users – pedestrians, motorcyclists, and cyclists – who comprise over half of all road traffic fatalities, worldwide (World Health Organization (WHO) 2013). These vulnerable groups make up an even greater proportion of road traffic fatalities in low-income countries at 57 %, compared to 51 % in middle-income countries and 39 % in high-income countries (WHO 2013). Factors that contribute to increased pedestrian and motorcyclist risk in LMICs include greater mixing of different road user types and limited or inadequately maintained infrastructure designed to accommodate different road users (Lagard 2007). For these reasons, it is vital that studies of RTI in motorizing nations assess and address the needs of these vulnerable groups.

Uganda ranks fifth out of 15 sub-Saharan African countries in highest DALYs due to RTI, according to the 2010 Global Burden of Disease study (Institute for Health Metrics and Evaluation 2013). Vulnerable road users suffer an estimated 68 % of all RTI fatalities in Uganda (WHO 2013). District hospitals provide first-line healthcare for the majority of Ugandans, and the WHO has emphasized the importance of strengthening emergency care at district hospitals as a key component of its strategy for improving surgical care in low-resource settings (Moroz & Spiegel 2014). Complete and accurate RTI data is vital for quality improvement in district hospital orthopedic care and has the potential to inform evidence-based road safety research and interventions for all, including the most vulnerable road users, in the districts of Uganda. Unfortunately, district hospital records and police recordsin Uganda tend to provide very little information on patient demographics and injury context and have significant levels of missing data preventing reliable analysis. Existing studies of road traffic injury in Uganda almost exclusively focus on tertiary referral centers (Andrews et al. 1999, Kobusingye and Lett 2000, Hsia et al. 2010, Jayaraman et al. 2015), with little known about patterns of musculoskeletal RTI at the district hospital level. In order to provide a more completedatasetthan has previously been available on orthopedic RTI in the districts of Uganda, we assessed patterns of musculoskeletal RTI and patient demographics by road user category at three Ugandan district-level hospitals for all patients presenting to these hospitals with musculoskeletal road traffic injuries identified on x-ray during the study period. With this data, we aim to guide further research on road traffic safety and to contribute knowledge to the global discussion of RTI at district hospitals in motorizing countries.

## Methods

This is a subgroup analysis of RTI patients from a larger musculoskeletal injury dataset. The full dataset analysis was previously reported by Kisitu et al. (2015). This study focuses on musculoskeletal injuries because these are reported by Mock and Meena (2008) to be the most common injuries at the district hospitals, which have the orthopedic units manned by orthopedic officers necessary to care for these injuries. Additionally, musculoskeletal injuries can easily be confirmed by x-ray, which is available at the district hospitals. This makes it easy for these types of injuries to be reliably diagnosed and analyzed unlike other forms of injuries which may need sophisticated diagnostic equipment for confirmation and interpretation of results.

Data for those patients suffering RTI are reported in this subgroup analysis in order to differentiate injury patterns by road user type. The inclusion criteria for the musculoskeletal injury study werepresentation to Mityana, Entebbe, or Nakaseke district hospitals from October 2013 to January 2014 with a musculoskeletal injury visible on x-ray. All patients meeting these criteria presenting to the study hospitals at any time of day or day of the week were recruited to participate in this cross-sectional study. The three hospitals located in central Uganda were selected based on their accessibility and the availability of a relatively high-volume orthopedic unit and an x-ray machine with adequate staffing. These hospitals comprise a range of settings, including the large town of Entebbe, the mid-sized town of Mityana along a major cross-country highway, and rural Nakaseke district.

The research team recruited and obtained informed consent for adults or assent for children after they had received the urgent care necessitated by their injuries. For fatally injured patients, consent to include patient demographic and injury data in the study was obtained from their next of kin. All patients received definitive care or, if necessary, stabilization and referral to a higher level of care, as per usual hospital protocols. The research team interviewed patients and/or next of kin, performed a physical exam, and ordered x-rays for radiographic diagnosis. All eligible patients consented to participate. In order to prevent bias, the questionnaire was pre-tested, research assistants received data collection training, questionnaires were reviewed regularly to limit the amount of missing data, and any low quality x-rays were repeated.

We assessed demographic characteristics, including age, sex, level of education, and occupation. We assessed the following categories of occupation: child, student, self-employed, employee of NGO/parastatal, civil servant, motorcycle taxi driver, housewife, subsistence farmer, unemployed, and other. Education was categorized as pre-schooling, student, less than or equivalent to secondary schooling, and post-secondary schooling. We also assessed road user type and the time of day at which the injury occurred. Each road user category includes both drivers and passengers of that mode of transportation. Location and type of injury and variables related to care-seeking behavior are not reported in this study, as the patterns for the RTI subset were very similar to patterns for the overall musculoskeletal injury dataset, which are reported in a separate article. We used R version 0.98.1103 for statistical analysis (R Core Team 2014). We report frequencies and percentages for the categorical variables in the overall RTI patient sample. We conducted chi-squared testing or fisher’s exact testing when cell counts were less than five for all comparisons. We applied the Bonferroni correction for multiple comparisons to the calculation of our *p*-values. We utilized the simple asymptotic method with continuity correction to calculate the 95 % confidence intervals for proportions.

We obtained study approval from the Department of Orthopedics and the School of Medicine Research and Ethics Committee at Makerere University, Kampala, Uganda. The Committee on Human Research of the University of California, San Francisco (UCSF) subsequently approved the involvement of the UCSF researchers in the data analysis. We sought permission from health facilities managers at the study hospitals prior to the beginning of data collection.

## Results

A total of 367 patients with musculoskeletal injuries were eligible to participate in the broader study of all musculoskeletal injuries from which this RTI subgroup analysis is derived. All of these patients consented and were included in the study. Data sheets for three patients were missing, leaving 364 patients for analysis. Among these 364 patients, 176 patients had suffered injuries from road traffic incidents and were included in this subgroup analysis.

**Fig. 1:**
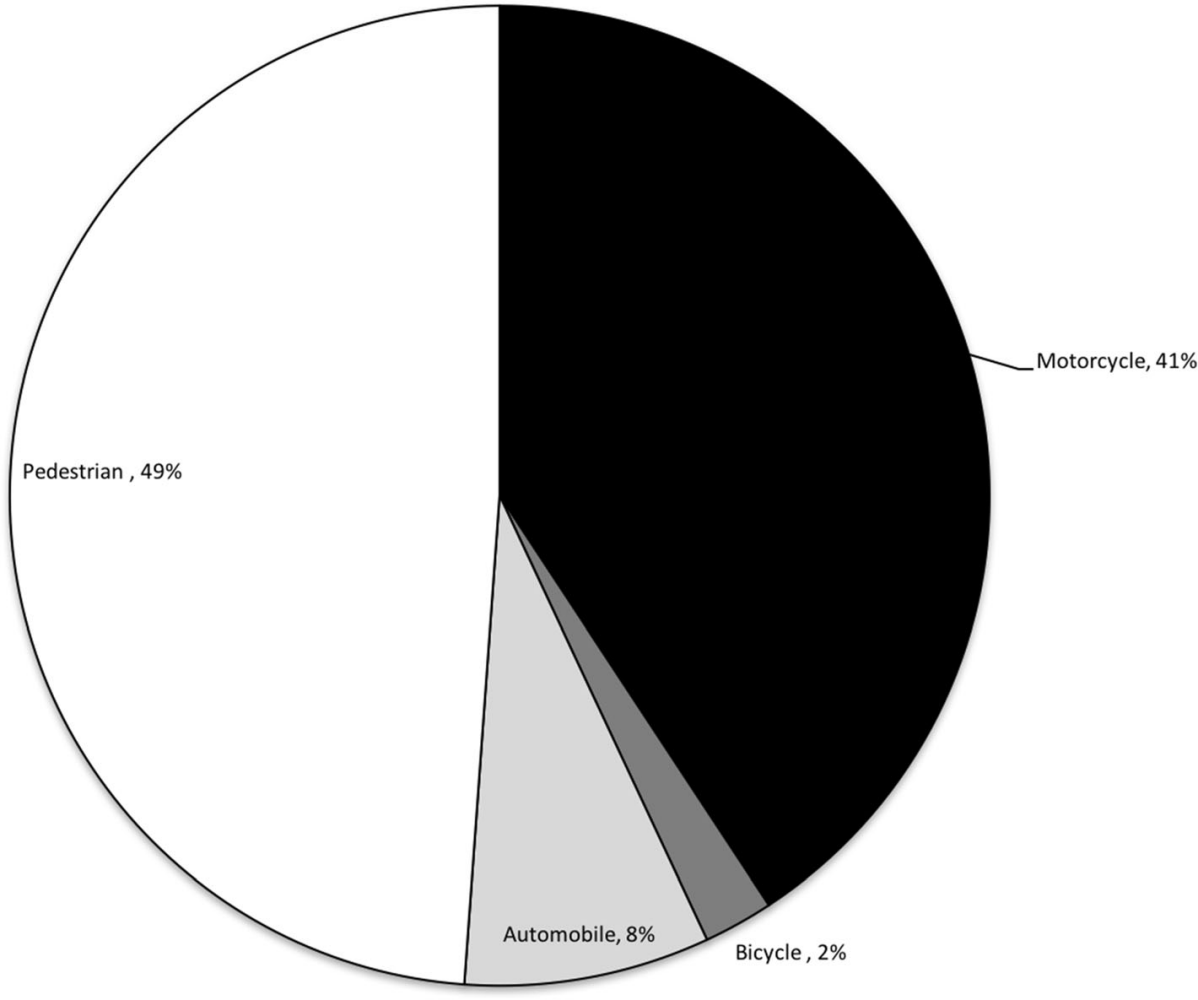
Distribution of road user types among the patient population (*n* = 172)

We found high proportions of vulnerable road users among the district hospital RTI patients, with 49 % (95 % CI 41–57 %) pedestrians, 41 % (95 % CI 33–49 %) motorcyclists, and 2 % (95 % CI 0–4 %) cyclists (Fig. 1). These proportions varied somewhat by hospital. Rural Nakaseke Hospital had 61 % (95 % CI 44–78 %) pedestrian injuries, 36 % (95 % CI 19–53 %) motorcycle injuries, 3 % (95 % CI 0–10 %) automobile injuries, and 0 % (95 % CI 0–1 %) bicycle injuries. The mid-sized town of Mityana reported 33 % (95 % CI 21–45 %) pedestrian injuries, 54 % (95 % CI 41–67 %) motorcycles injuries, 8 % (95 % CI 1–15 %) automobile injuries, and 5 % (95 % CI 0–11 %) bicycle injuries. The large town of Entebbe reported 56 % (95 % CI 44–68 %) pedestrian injuries, 32 % (95 % CI 21–43 %) motorcycle injuries, 11 % (95 % CI 3–19 %) automobile injuries, and 1 % (95 % CI 0–4 %) bicycle injuries.

The RTI patients were 71 % (95 % CI 64–78 %) male and only 29 % (95 % CI 22–36 %) female (Table 1). Thirty-one percent (95 % CI 21–41 %) of pedestrians were less than 15 years old, and pedestrians were significantly more likely than motorcyclists to be in the <15-year-old age category (*p* < 0.001). Thirty-nine percent (95 % CI 27–51 %) of motorcyclists were between the ages of 15 and 29 years. There was no significant difference in the proportions of males and females in the different road user categories. Although both pedestrians and motorcyclists were most commonly non-students with less than a post-secondary education (48 % (95 % CI 37–59 %) and 74 % (95 % CI 63–85 %), respectively), motorcyclists were significantly more likely to report this educational level than pedestrians (*p* = 0.03). Student was the most common occupational category among pedestrians (33 % (95 % CI 22–44 %) of pedestrians v 10 % (95 % CI 2–18 %) of motorcyclists, *p* = 0.02), while the most common occupations among motorcyclists were “self-employed” (42 % (95 % CI 30–54 %) of motorcyclists, 29 % (95 % CI 19–39 %) of pedestrians, *p* = 1) and subsistence farmer (19 % (95 % CI 9–29 %) of motorcyclists, 15 % (95 % CI 7–23 %) of pedestrians, *p* = 1). Demographic comparisons between the other groups of road users did not achieve statistical significance, likely due to small sample sizes.

**Table 1:** Demographic characteristics by type of road user (frequency (percent))

	RTI Patients		Pedestrians		Motorcyclists		*P* value Comparing pedestrians to motorcyclists^a^
Characteristic	*n* = 176	% (95 % CI)	*n* = 84	% (95 % CI)	*n* = 70	% (95 % CI)	
Age (years)							
0–14	29	16 % (10–22 %)	25	31 % (21–41 %)	3	5 % (0–11 %)	<0.001
15–29	56	32 % (25–39 %)	22	27 % (17–37 %)	26	39 % (27–51 %)	1
30–44	42	24 % (17–31 %)	19	23 % (13–33 %)	16	24 % (13–35 %)	1
45–59	30	17 % (11–23 %)	10	12 % (4–20 %)	17	26 % (15–37 %)	1
> 59	11	6 % (2–10 %)	5	6 % (0–12 %)	4	6 % (0–12 %)	1
Sex							
Male	125	71 % (64–78 %)	57	68 % (57–79 %)	52	74 % (63–85 %)	1
Female	51	29 % (22–36 %)	27	32 % (21–43 %)	18	26 % (15–37 %)	
Education							
Pre-Schooling	4	2 % (0–4 %)	3	4 % (0–9 %)	1	1 % (0–4 %)	1
Student	40	23 % (16–30 %)	27	33 % (22–44 %)	7	10 % (2–18 %)	0.02
≤ Secondary Schooling	100	57 % (49–65 %)	39	48 % (37–59 %)	51	74 % (63–85 %)	0.03
Post-Secondary Schooling	27	15 % (09–21 %)	12	15 % (7–23 %)	10	14 % (5–23 %)	1
Occupation							
Child	4	2 % (0–4 %)	3	4 % (1–9 %)	1	1 % (0–4 %)	1
Student	39	23 % (16–30 %)	27	33 % (22–44 %)	7	10 % (2–18 %)	0.02
Self Employed	58	34 % (27–41 %)	24	29 % (19–39 %)	29	42 % (30–54 %)	1
Employee of NGO/Parastatal	15	9 % (4–14 %)	8	10 % (3–17 %)	6	9 % (2–16 %)	1
Civil Servant	5	3 % (0–6 %)	0	0 % (0–1 %)	4	6 % (0–12 %)	0.83
Motorcycle Taxi Driver	6	3 % (0–6 %)	1	1 % (0–4 %)	4	6 % (0–12 %)	1
Housewife	4	2 % (0–4 %)	2	2 % (0–6 %)	2	3 % (0–8 %)	1
Subsistence Farmer	26	15 % (9–21 %)	12	15 % (7–23 %)	13	19 % (9–29 %)	1
Unemployed	4	2 % (0–4 %)	3	4 % (1–9 %)	1	1 % (0–4 %)	1
Other	7	4 % (1–7 %)	2	2 % (0–6 %)	2	3 % (0–8 %)	1

^a^Bonferroni-corrected chi-squared or fisher’s exact test comparing pedestrians to motorcyclists

**Fig. 2:**
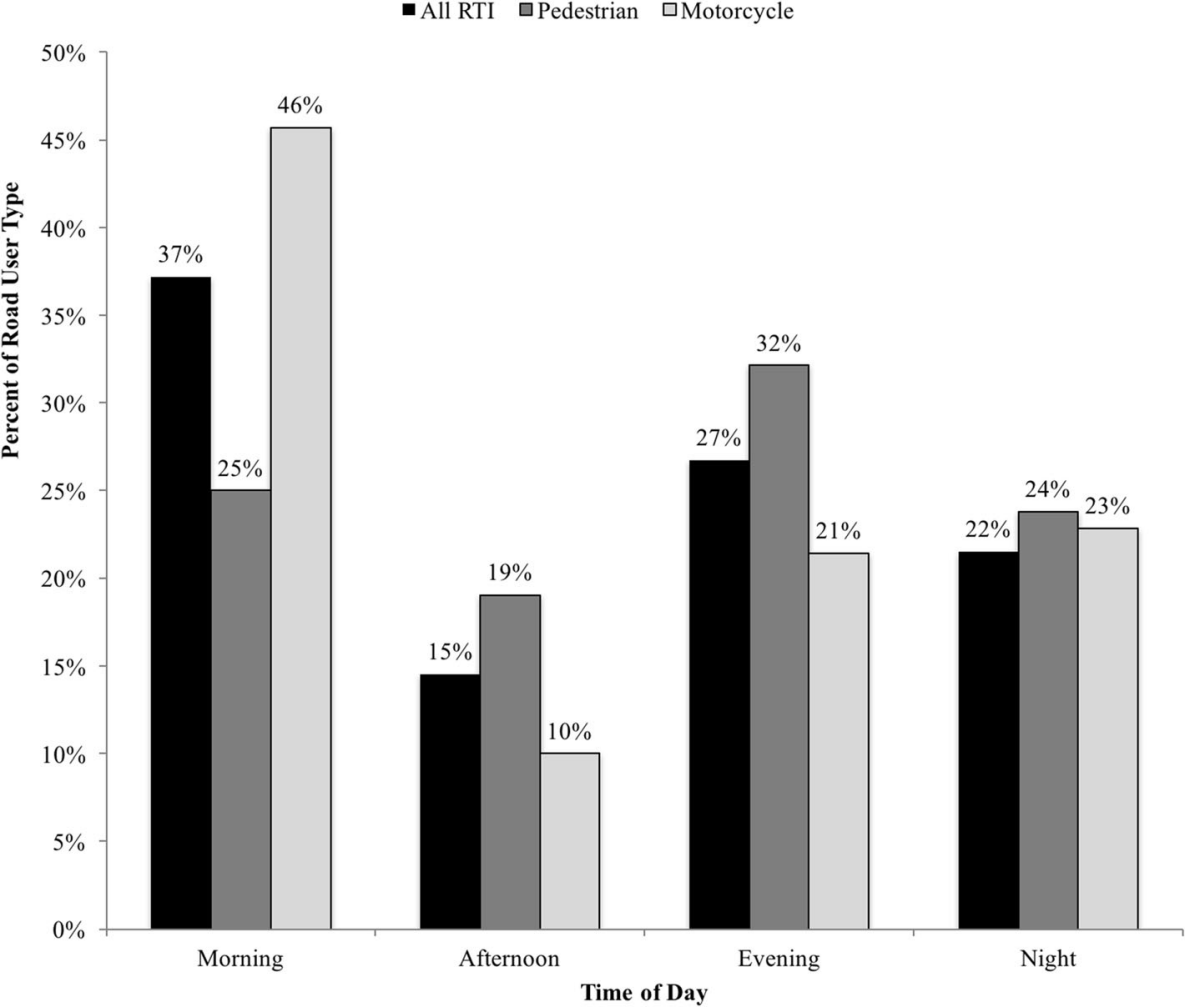
Injury time of day^a^ by road user type (*n* = 172). ^a^Time at which injury occurred.

Injuries most commonly occurred during morning (37 %; 95 % CI 30–44 %) and evening (27 %; 95 % CI 20–34 %) hours (Fig. 2). The most common time for motorcyclist injuries was also in the morning (46 %; 95 % CI 34–58 %), followed by the nighttime (23 %; 95 % CI 12–34 %), and evening (21 %; 95 % CI 11–31 %). Pedestrian injuries were more evenly distributed throughout the day, but were most common in the evening (32 %; 95 % CI 21–43 %).

## Discussion

Data-driven policies for the prevention and management of musculoskeletal injuries resulting from RTI are essential as sub-Saharan African nations continue to motorize. Data from district hospitals in sub-Saharan Africa is lacking but has the potential to contribute valuable information to two important World Health Organization initiatives. First, the WHO Global Initiative for Emergency and Essential Surgical Care emphasizes the importance of focusing surgical and trauma systems capacity-building efforts at the district hospital level, where the majority of the population of many African nations receive care (Moroz & Spiegel 2014). Second, the WHO’s *Global Status Report on Road Safety* emphasizes the need to target road safety interventions toward pedestrians, motorcyclists, bicyclists, and other vulnerable road users (WHO 2013). This study found that RTI patients presenting to these three district hospitals for care of musculoskeletal injuries were primarily vulnerable road users. Commonly injured demographic groups by road user category included student pedestrians and motorcyclists with less than a post-secondary education. The morning hours were the most common time of injury for all RTI patients as well as for motorcyclists, while pedestrians were most commonly injured in the evening. The high proportion of working-age men in the study demonstrates the impact of RTI on the most economically productive sector of Ugandan society. This injury surveillance study suggests specific avenues for future research focused on musculoskeletal road traffic injury prevention and management targeted to these most commonly injured demographic groups. These results also demonstrate the importance of district hospitals in caring for vulnerable road users and suggest the key role that district hospitals could playin strategies for achieving WHO goals for road safety and trauma systems strengthening.

The results of this study emphasize the importance of safety interventions aimed at protecting vulnerable road users. Among the musculoskeletal RTI patients included in the study, 49 % (95 % CI 41–57 %) were pedestrians, and 41 % (95 % CI 33–49 %) were motorcyclists. Previous studies based primarily at Mulago Hospital and other hospitals in Kampala have found lower proportions of vulnerable road users among all RTI patients as follows: 43.5 % pedestrians (Andrews et al. 1999), 38 % pedestrians and 22 % cyclists (Kobusingye et al., 2002), 33.4 % pedestrians and 24.4 % cyclists (Kobusingye and Lett 2000), 30 % pedestrians (Hsia et al. 2010), and 14.5 % crashes involving a motorcycle (Jayaraman et al. 2015), respectively. This study thus suggests that vulnerable road users may make up a larger percentage of district hospital RTI patients with musculoskeletal injuries compared with RTI patients seen at hospitals in Kampala.

There are several possible explanations for this discrepancy. The increased proportion of motorcyclists in the present study compared to these previous studies from Kampala may be partially explained by increasing motorcycle use in Uganda during the past decade. Nonetheless, given that the Ugandan 2012 Road User Satisfaction Survey estimates that motorcyclists comprise only 19 % of all vehicles in Uganda (CrossRoads 2012), the high proportion of motorcyclists among district hospital RTI patients likely involves the increased risk of this mode of transportation, as well as increased motorcycle use. Furthermore, according to Ugandan police records from 2010, pedestrians and motorcyclists comprised only 41 and 17 % of road traffic fatalities, respectively (WHO 2013), suggesting that these vulnerable groups are overrepresented among district hospital patients with musculoskeletal injuries compared to all fatal crash victims. Neither this police data nor the previously mentioned RTI studies from Kampala provide perfect comparisons for the data in this study given that they analyzed fatalities and all RTI injuries, rather than musculoskeletal injuries. However, injury severity tends to be higher among pedestrians and motorcyclists compared to automobile occupants (McGreevy et al. 2014), and a study of RTI patients from Kenya found higher rates of head, thorax, and abdominal injuries among pedestrians and motorcyclists compared to automobile occupants (Osoro et al. 2011). For these reasons, we would not expect these vulnerable road users to be so overrepresented among patients with isolated orthopedic injuries compared to all RTI victims.

A likely explanation for the apparent overrepresentation of vulnerable road users among district hospital patients is that those who use less expensive modes of transportation may have fewer financial resources available to them and may choose to seek care at the more affordable district hospitals. Private hospitals are generally more expensive, and referral centers are hard for the poor in more remote areas to access. A high proportion of study patients were children, another vulnerable group, which may also contribute to the high proportion of pedestrians in the study. Given that 92 % of all district hospital musculoskeletal injuries due to RTI were among pedestrians, motorcyclists, or cyclists, the results of this study suggest that Ugandan district hospitals may care for a disproportionate share of vulnerable road users. Because addressing the risk of morbidity and mortality among vulnerable road users is a key component of the WHO strategy for decreasing the disease burden from RTI (Peden et al. 2004), these results demonstrate the importance of strengthening musculoskeletal care at the district hospital level in order to achieve this goal.

Demographic differences between motorcyclists and pedestrians demonstrate commonly injured sub-populations among these vulnerable road users. Although we do not know whether these groups have greater risk or greater exposure, their high numbers suggest that they could be important target groups for safety interventions. Motorcyclists and pedestrians exhibit differences in their age and education, though much of the difference in educational attainment is likely due to the differences in the age distributions of these two populations. Motorcyclists tended to be adult males with less than a post-secondary education who most commonly listed their occupations as either self-employed or subsistence farmer. Safety interventions such as educational initiatives or promotion of helmet and reflective clothing use could be targeted to these groups. Among pedestrians, two of the largest groups were children younger than age 15 (31 %; 95 % CI 21–41 %) and students (33 %; 95 % CI 22–44 %). Safety interventions aimed at protecting children in transit to and from school could help decrease injuries among this sub-group.

Pedestrians and motorcyclists also exhibited differences in the timing of their injuries. The morning hours were the most common time for all RTI (37 %; 95 % CI 30–44 %) as well as for motorcyclist injuries (46 %; 95 % CI 34–58 %). Pedestrian injuries were more evenly spread throughout the day but were most common in the evening (32 %; 95 % CI 21–43 %). Multiple studies from the United States comparing pedestrian injury rates before and after daylight saving time transitions have implicated decreased lighting in the evening hours as a significant risk factor for pedestrian injury (Ferguson et al. 1995; Sullivan & Flannagan 2002). Poor visibility is thus a likely contributing factor to the high rates of evening pedestrian injury found in this study. Overall, the results of this study suggest that resources for road safety enforcement might be best allocated in this region during the morning and evening hours and that interventions aimed at increasing visibility after dusk should be considered.

One limitation of hospital-based studies is that patterns of injury and care-seeking behavior may differ among the overall population compared with the hospital patient population. Therefore, demographic groups that choose to seek care elsewhere are not represented in this analysis. Injuries either too minor for patients to seek care or major enough to cause pre-hospital fatalities are also not captured by hospital-based records. A further limitation is the availability of diagnostic equipment in the district hospitals; only injuries evident on x-ray were analyzed and only hospitals with functional x-ray machines were included. Despite these limitations, this is to our knowledge the only study of RTI in district hospitals in Uganda.

This cross-sectional study helps to target future research on RTI safety interventions in the districts of central Uganda. Particular areas of focus could include safety of school children pedestrians, increased police enforcement during morning and evening commutes, and increased roadway and road user visibility during evening hours. The results of this study also suggest that Ugandan district hospitals may care for a disproportionate share of vulnerable road users compared to hospitals in larger cities such as Kampala. District hospitals must receive sufficient funding, resources, and staffing in order to ensure that all Ugandans, including the most vulnerable road users, receive appropriate musculoskeletal care.

## Conclusions

This descritive analysis of at-risk road users and times of day that road traffic injuries occur can help inform and direct prevention efforts in regions of Uganda outside of the large city of Kampala.

## Abbreviations

DALYs: Disability-adjusted life years
LMICs: Low- and middle-income countries
RTI: Road traffic injury
UCSF: University of California, San Francisco
WHO: World Health Organization
